# Two decades of antenatal and delivery care in Uganda: a cross-sectional study using Demographic and Health Surveys

**DOI:** 10.1186/s12913-018-3546-3

**Published:** 2018-10-04

**Authors:** Lenka Benova, Mardieh L. Dennis, Isabelle L. Lange, Oona M. R. Campbell, Peter Waiswa, Manon Haemmerli, Yolanda Fernandez, Kate Kerber, Joy E. Lawn, Andreia Costa Santos, Fred Matovu, David Macleod, Catherine Goodman, Loveday Penn-Kekana, Freddie Ssengooba, Caroline A. Lynch

**Affiliations:** 1 London School of Hygiene and Tropical Medicine, Keppel Street, London, WC1E 7HT UK; 2 Institute of Tropical Medicine, Nationalestraat 155, 2000 Antwerp, Belgium; 3 School of Public Health, Makerere University, P.O Box 7072, Kampala, Uganda; 4 Saving Newborn Lives, Save the Children, 899 North Capitol Street, Suite 900, Washington, DC, 20002 USA; 5 Indigenous & Global Health Research Group, Department of Medicine, University of Alberta, University Terrace, 8303-112 Street, Edmonton, AB T6G 2T4 Canada; 6 School of Economics, Makerere University Kampala, Uganda and Policy Analysis & Development Research Institute (PADRI), Kampala, Uganda

**Keywords:** Maternal health, Antenatal care, Childbirth location, Delivery care, Facility delivery, Public sector, Private sector, Content of care, Quality of care, Socio-economic inequalities

## Abstract

**Background:**

Uganda halved its maternal mortality to 343/100,000 live births between 1990 and 2015, but did not meet the Millennium Development Goal 5. Skilled, timely and good quality antenatal (ANC) and delivery care can prevent the majority of maternal/newborn deaths and stillbirths. We examine coverage, equity, sector of provision and content of ANC and delivery care between 1991 and 2011.

**Methods:**

We conducted a repeated cross-sectional study using four Uganda Demographic and Health Surveys (1995, 2000, 2006 and 2011).Using the most recent live birth and adjusting for survey sampling, we estimated percentage and absolute number of births with ANC (any and 4+ visits), facility delivery, caesarean sections and complete maternal care. We assessed socio-economic differentials in these indicators by wealth, education, urban/rural residence, and geographic zone on the 1995 and 2011 surveys. We estimated the proportions of ANC and delivery care provided by the public and private (for-profit and not-for-profit) sectors, and compared content of ANC and delivery care between sectors. Statistical significance of differences were evaluated using chi-square tests.

**Results:**

Coverage with any ANC remained high over the study period (> 90% since 2001) but was of insufficient frequency; < 50% of women who received any ANC reported 4+ visits. Facility-based delivery care increased slowly, reaching 58% in 2011. While significant inequalities in coverage by wealth, education, residence and geographic zone remained, coverage improved for all indicators among the lowest socio-economic groups of women over time. The private sector market share declined over time to 14% of ANC and 25% of delivery care in 2011. Only 10% of women with 4+ ANC visits and 13% of women delivering in facilities received all measured care components.

**Conclusions:**

The Ugandan health system had to cope with more than 30,000 additional births annually between 1991 and 2011. The majority of women in Uganda accessed ANC, but this contact did not result in care of sufficient frequency, content, and continuum of care (facility delivery). Providers in both sectors require quality improvements. Achieving universal health coverage and maternal/newborn SDGs in Uganda requires prioritising poor, less educated and rural women despite competing priorities for financial and human resources.

**Electronic supplementary material:**

The online version of this article (10.1186/s12913-018-3546-3) contains supplementary material, which is available to authorized users.

## Background

By the end of the Millennium Development Goals (MDG) period in 2015, Uganda met its goal for reducing under-5 child mortality, with a 4.9% average annual reduction in the death rate between 1990 and 2015 [1]. During this time, Uganda’s maternal mortality ratio (MMR) declined from 687 to 343 per 100,000 live births at an annual rate of 2.8%, which accelerated after 2000 [2]. However, the country did not meet its MDG MMR target of 200 per 100,000 live births and the absolute number of maternal deaths per year remained around 6000. This was partly due to population growth, which more than doubled the population size between 1990 and 2014 from 17.4 to 37.8 million [2, 3]. Uganda is one of the high-burden countries contributing the highest absolute numbers of maternal deaths [4] and deliveries when the woman was alone with no one present [5] in sub-Saharan Africa; stillbirths and neonatal deaths are also high [6].

Antenatal care (ANC) and skilled delivery care can prevent maternal and perinatal deaths [7–11], and their coverage is routinely used to monitor progress toward reducing maternal and neonatal mortality. The role of routine focused ANC in monitoring pregnancy is three-fold: to prevent conditions that may have unfavourable effects on the health of the mother and child, to treat complications and to provide information about pregnancy, childbirth and the postnatal period to the mother [12, 13]. The majority of maternal and perinatal deaths occur around the time of labour, delivery and the immediate postpartum period [14, 15]. Effective interventions exist to prevent, manage and treat virtually all the causes of maternal complications; most of these are included in Ugandan health policies, but a considerable gap between policy and implementation exists. One of the strategies for improvement in the survival and health of women and their newborns is to ensure deliveries are conducted by skilled birth attendants (SBA) [16, 17].

The Countdown to 2015 Uganda country profile highlighted that after a long period of virtually no increase in the percentage of births occurring with a SBA, this indicator increased substantially from 42% in 2006 to 57% in 2011. However, large unmet need for maternal and newborn care remained and the socio-economic inequities in maternal care coverage were the widest among all presented indicators of maternal, newborn and child health [18].

The Sustainable Development Goals (SDG) aim to reduce the global MMR to below 70 per 100,000 live births. Achieving this goal as well as universal coverage with good quality ANC and delivery care will require considerable resources and policy prioritisation. The high population growth in Uganda (3% annually and a total fertility rate of 6.2 in 2011) [3, 19] means that Uganda needs to provide more services year-on-year just to maintain existing coverage levels. Equity analyses can help identify the type of women that are excluded from receiving these lifesaving services. This paper comprehensively assesses the coverage, equity, sector of provision and content of ANC and maternal care between 1991 and 2011, and can thus contribute to the policy discussions on strategies toward achieving SDG targets including the roles of public and private providers in maternal care provision.

Additional file 1: Figure S1 presents a brief timeline of Uganda’s policies on maternal health over the period under consideration, as well as national goals and strategies related to maternal health [19–24]. It shows increasing attention to maternal health over time and the presence of large programmes aimed at improving maternal/newborn health outcomes. One key health policy during this period was the removal of user fees for all services in 2001 [25, 26]. Despite this major policy commitment to remove financial barriers to public health services, in practice, households continued to pay nearly 50% of the total costs for reproductive health services out of pocket [27], largely due to expenses incurred using private providers.

Health facilities in Uganda are categorised into six levels, ranging from national referral hospitals, responsible for providing the highest level specialist care, to level II health centres, responsible for providing preventive and promotive outpatient and outreach services [28]. Facility-based care is complemented by the lowest level of health service provision – health promotion activities at the community level [29]. While the government provides formal care at all levels, the private sector is more diverse and includes a range of formal, informal, for-profit, not-for-profit, and faith-based providers [25, 30, 31]. The private sector is a significant contributor to health service delivery in Uganda generally, with 45% of the country’s health facilities owned by non-state actors [28]. However, fewer than 20% of private health facilities are classified as level III health centres or higher, the minimum level at which maternal health services are to be provided, and the majority of these are not-for-profit facilities [28, 29]. A number of interventions aimed at capitalising on the presence of the private sector have been implemented in Uganda over the years, some with an express aim to increase coverage among hard-to-reach groups and to alleviate the undue financial burden of reproductive health services (e.g., voucher schemes) [32–36]. Though these interventions were often evaluated separately, the contribution of the private sector to maternal and reproductive health in Uganda has not been assessed comprehensively over time.

This paper’s main objective is to aid the formulation of future strategies by describing the historical development and recent levels of utilisation of maternal care in Uganda, focusing on ANC and delivery care. We used the data collected by the Demographic and Health Surveys (DHS), to address three specific aims. The first aim of the study is to describe, between 1991 and 2011: (1) the coverage of ANC and delivery care, and (2) the equity of ANC and delivery care coverage. The second aim is to describe the role of the public and private (non-governmental) sectors in delivering ANC and delivery care over time and to assess its equity. The final aim of the paper is to examine the content of ANC and delivery care reported on the most recent survey, and to compare across the two sectors.

## Methods

### Data

The DHS are cross-sectional nationally representative surveys, usually covering 5000 to 30,000 households. In the DHS, standard model questionnaires are used but can be adapted by each country. They use standard model questionnaires, which countries can adapt by adding optional modules, questions or response options. The surveys include questions on household and individual characteristics, fertility and family planning, maternal and child health and details on antenatal and delivery care. The sampling design is a multi-level cluster survey, which often oversamples certain areas. We used data from the 1995, 2000–2001, 2006, and 2011 Uganda DHS rounds [19, 22–24]. We used demographic data on population size and crude birth rate from the United Nations Population Division to make population-level projections [3].

### Population

The analysis sample was drawn from all women age 15–49 interviewed in the sampled households. All women aged 15–49 with need for ANC or delivery care services – defined as having one or more live births in each survey’s 5-year recall period – were included in this analysis (samples for all analyses in this paper are described in Additional file 2: Table S1). Although information was collected from these women on delivery care for all births during the recall period, questions about ANC were only asked for the most recent birth. We conducted a sensitivity analysis, which showed that indicators of coverage for delivery care were broadly comparable irrespective of whether all births or only the most recent birth was the analysis sample (estimates were somewhat lower for all births compared to most recent births, but 95% confidence intervals overlapped in all surveys, Additional file 2: Table S2). For purposes of comparability between ANC and delivery care indicators, we limited our analysis to the most recent live birth (one birth per woman) in the recall period. We present cross-sectional estimates of each indicator for the 5-year recall period of each surveys; for example, the 2011 survey estimates are presented as for the period 2007–2011.

### Indicators and definitions

#### Coverage indicators

This study explored five indicators related to coverage of ANC and delivery care services.

Any ANC: Women were asked how many ANC visits they received for their most recent birth and to list all ANC locations (i.e., providers) and, separately, to provide all cadres of health professional from whom they received this care. We defined receipt of any ANC as reporting one or more ANC visits during pregnancy and reporting that at least one cadre of medical professional was seen during ANC, regardless of the location where care was received. We considered medical professionals to be doctors, nurses/midwives, auxiliary midwives, medical assistants/clinical officers, and nursing aides. All other persons, including traditional birth attendants, were not considered medical professionals. If either the number of ANC visits or the type of cadre was missing, women were considered as not having received any ANC.

Recommended ANC: 4+ ANC visits during pregnancy and at least one medical professional among the list of cadres seen during ANC, according to World Health Organization (WHO) recommendations at the time of the surveys [37]. Women who received fewer than four visits (including none) and those who received 4+ visits but none from a medical professional were classified as having suboptimal ANC. We also conducted sensitivity analyses further restricting the definition of recommended ANC to women who initiated ANC in first trimester of pregnancy and those who received 8+ visits [38].

Facility delivery care: Respondents were asked to indicate the location of their most recent delivery and list everyone who assisted with the delivery. We do not wish to imply that women who delivered in health facilities necessarily received good quality intrapartum care, but that the location fulfilled the minimum requirements for availability of such care [39]. Births with missing information on location were assumed not to have occurred in a health facility. Women who did not deliver in a facility were classified as receiving suboptimal delivery care; this includes women who reported assistance from a SBA in a non-facility location [40]. Over 99% of women with facility births in the sample reported being attended by a SBA (a doctor, nurse/midwife, auxiliary midwife, or medical assistant/clinical officer), and a small percentage of births (5.2% on the 2011 survey) were conducted with an SBA in a home or other non-facility location.

Caesarean section: The proportion of births occurring by caesarean section can be a good, albeit crude, indicator of potential unmet need for emergency delivery care, or vice versa, of unnecessary surgical intervention [41–43]. Women were considered to have delivered by caesarean section if they gave birth in a health facility and indicated having had a caesarean section. The few cases of reported caesarean sections occurring outside of a health facility and births with missing information for type of delivery were re-coded as vaginal deliveries.

Complete maternal care: To assess co-coverage (receipt of multiple services) of ANC and delivery care we considered women who received recommended ANC and delivered in a facility to have received complete care for maternal services. Again, we do not intend to imply that women with complete maternal care necessarily received good quality pregnancy, delivery and postpartum care; instead we use it as a proxy indicator following global guidelines given data available from the surveys. Women who did not meet both conditions were classified into one of five categories: (a) no ANC and suboptimal delivery care, (b) some ANC and suboptimal delivery care, (c) complete ANC and suboptimal delivery care, (d) no ANC and facility delivery care, or (e) some ANC and facility delivery care.

#### Sector of care

We assessed the contribution of each sector to provision of care for women who received any ANC and facility delivery care. We categorized delivery care as received in a public or private (all non-state providers including for-profit and not-for-profit facilities) sector facility; this information was collected in all four surveys (Additional file 2: Table S3). Women could list more than one provider of ANC services. We categorized ANC as being received in a public sector facility, private sector facility, a combination of both public and private sector facilities, or only at home/other locations; this information was only collected on the two most recent surveys (2006 and 2011). Among women who accessed ANC from multiple locations, those who reported receiving ANC at one or more public sector facility types were classified as public sector, regardless of whether they also received additional ANC in a home location. Similarly, those who reported receiving ANC at one or more private sector facility types were classified as private sector, regardless of whether they additionally received ANC from a home location.

#### Equity indicators

We assessed the indicators of coverage and co-coverage for the earliest and most recent surveys (1995 and 2011) according to four socio-economic characteristics: (a) geographical zone (Central, Eastern, Northern, and Western), (b) residence (urban, rural), (c) women’s education level (no education, any primary education, and any secondary or higher education), and (d) wealth quintiles (using standard method of categorizing asset ownership into five equally sized groups) [44].

The 1995 and 2000–2001 surveys excluded some districts due to security risks and were therefore not nationally representative [22, 24]. Moreover, the standard administrative regions are not geographically comparable over time due to increase in the number of districts. In order to facilitate geographic comparisons, we used the lowest common subnational geographic units to create four geographical zones with the same boundaries across all four surveys. These are comprised of the following regions from the 2011 Uganda DHS: Central (Central 1, Central 2, and Kampala), Eastern (Eastern and East Central), Northern (West Nile, North, and Karamoja), and Western (Western and Southwest). The districts missed by the 1995 and 2000–2001 surveys were all in the Northern zone.

#### Content of care by sector

We examined the content of ANC and delivery care using data from the most recent survey (2011). We were unable to explore changes in the content of care over time because care components were captured inconsistently on the earlier surveys.

Content of ANC care among women who received recommended ANC: The survey asked women whether they received eight specific components of ANC, including whether they were weighed; had blood pressure measured; had urine or blood samples taken; were told about pregnancy complications; took iron supplements, drugs for intestinal parasites, and malaria prophylaxis during pregnancy. We assigned a score to each woman, calculated as the percentage of eight possible ANC components.

Content of delivery care among women who delivered in health facilities: Women were asked about four aspects of care received at the time of their most recent birth, including whether they initiated breastfeeding within 1 h of giving birth; whether the baby was weighed; the length of stay (LOS) in the facility after delivery; and whether anyone checked on the woman’s health before she was discharged from the facility. Responses to the LOS question were converted into hours based on methodology used previously [45] and characterised as too short according to type of delivery (vaginal < 24 h and caesarean < 72 h). We created a score as a percentage from four possible components.

In examining each component of care, we considered the response “Don’t know” and missing values (< 3% of women in any single component) not to have received the particular component. In calculating the content of care score, women with such values were excluded from analysis (7.3% of recommended ANC users and 2.4% of facility-based delivery users).

### Data analysis

Our analysis of coverage of ANC and delivery care estimated the percentage of women who received some ANC, recommended (4+ visits) ANC, facility-based delivery care, caesarean section rate, and complete maternal care for their most recent birth. We estimated coverage indicators across time for each survey, and examined inequity of coverage over time by wealth, education, residence and geographic region. We also estimated the total number of births that received the five elements of care coverage overall, and by sector in the 5-year period before each survey. We estimated the percentage of women receiving each content of care component on the 2011 survey by sector.

Using chi2 *p*-values, we statistically assessed the difference in the five coverage indicators over time (change between 1995 and 2011), between the extreme categories of wealth (richest versus poorest quintiles), education and residence (on the 1995 and the 2011 surveys). We also assessed the difference in percentages of women receiving each component of care between the public and private sector on the 2011 survey.

Analyses were conducted using Stata/SE 14 (College Station, Texas, USA). All reported estimates were adjusted to account for clustering and stratification in the survey sampling design. We weighted the data using the individual weights provided with the DHS datasets.

## Results

### Coverage and co-coverage of ANC and delivery care over time

**Fig. 1:**
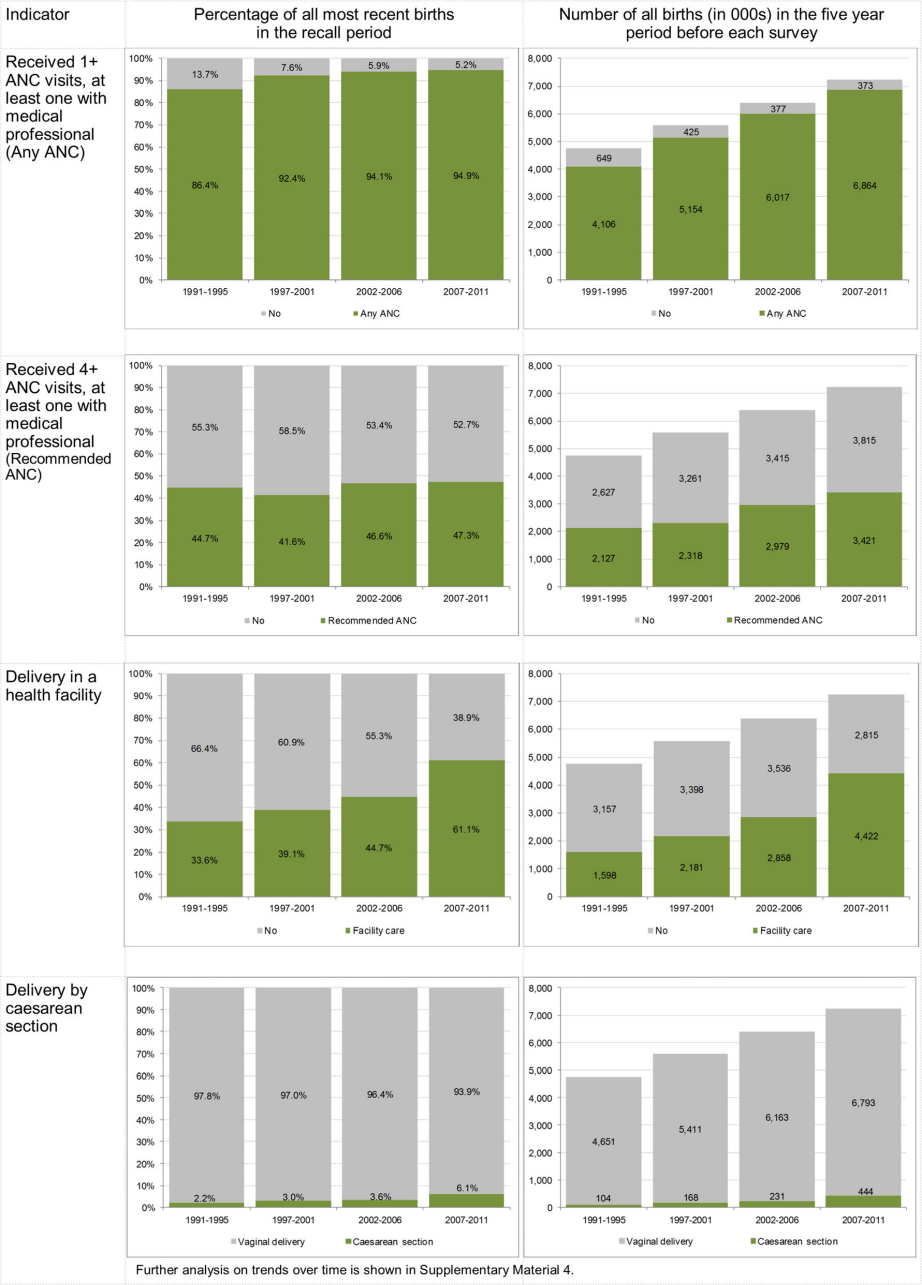
Coverage indicators for most recent live births, by survey period

Between 1991 and 2011, the average annual number of births occurring in Uganda increased from 950,000 to nearly 1.5 million (Table 1). The coverage with any ANC was high over the period under analysis. It increased significantly (*p* < 0.001) from 86.4% (1991–1995) to 94.9% (2007–2011), as shown in Fig. 1 and Additional file 2: Table S4. The number of births for which ANC services were provided increased from 4.1 million (1991–1995) to 6.9 million (2007–2011). However, the proportion of births receiving recommended ANC stagnated below 50% during the entire period under consideration, with no significant increase between 1995 and 2011 surveys. Further analysis of timing of ANC showed that in 2011, only 33.1% of women with recommended ANC initiated ANC in the first trimester of pregnancy. The percentage of women who met the new WHO ANC guidelines (8+ visits at least one with a medical professional, start in the first trimester) was negligible and declined over time (2.8% in 1995 and 1.1% in 2011).

**Table 1:** Number of births by survey recall period

Survey	Years in recall period	Number of births in five-year period before survey	Average annual number of births
1995	1991–1995	4,754,595	950,919
2001	1997–2001	5,579,012	1,115,802
2006	2002–2006	6,394,207	1,278,841
2011	2007–2011	7,236,646	1,447,329

Calculated based on annual population estimates (1991–2011) and quinquennial crude birth rate estimates from United Nations Department of Economic and Social Affairs Population Division: World Population Prospects: The 2015 Revision

Figure 1 and Additional file 2: Table S4 show that the percentage of births with facility delivery care increased significantly from 33.6 to 61.1% between 1991 and 1995 and 2007–2011(*p* < 0.001). The largest increase occurred between the two most recent surveys. The absolute number of births occurring in facilities rose nearly three-fold from 1.6 million in 1991–1995 to 4.4 million in 2007–2011. Despite this increase, 2.8 million live births were delivered outside of health facilities in 2007–2011. The caesarean section rate significantly increased from 2.2% in 1991–1995 to 6.1% in 2007–2011 (p < 0.001). An estimated 444,000 caesarean sections were performed in 2007–2011, a four-fold increase from just over 100,000 in 1991–1995.

**Fig. 2:**
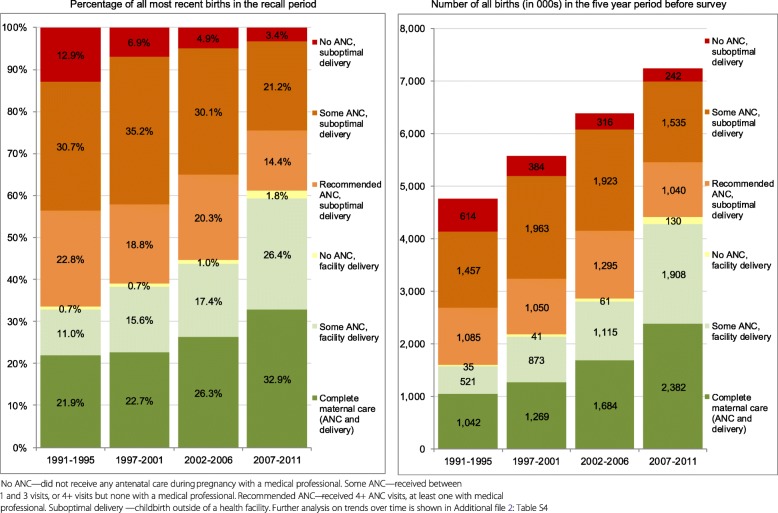
Co-coverage of ANC and delivery care for most recent live births, by survey period

When the complete maternal care package was assessed (combining recommended ANC and facility delivery), coverage increased significantly from 21.9% of births in 1991–1995 to 32.9% in 2007–2011 (p < 0.001, Fig. 2 and Additional file 2: Table S4). The absolute number of births that received complete care more than doubled, from 1.0 to 2.4 million between 1991 and 1995 and 2007–2011. The proportion of women who did not receive any maternal care declined from 12.9% in 1991–1995 to 3.4% in 2007–2011, corresponding to a decline from 614,000 to 242,000 births without any ANC and facility delivery care. A large percentage of women received some care from the health system without attaining complete care, and this percentage remained fairly constant (65.2 and 63.7%, respectively) between the 1991–1995 and 2007–2011 time periods.

### Equity of coverage and co-coverage of ANC and delivery care over time

**Fig. 3:**
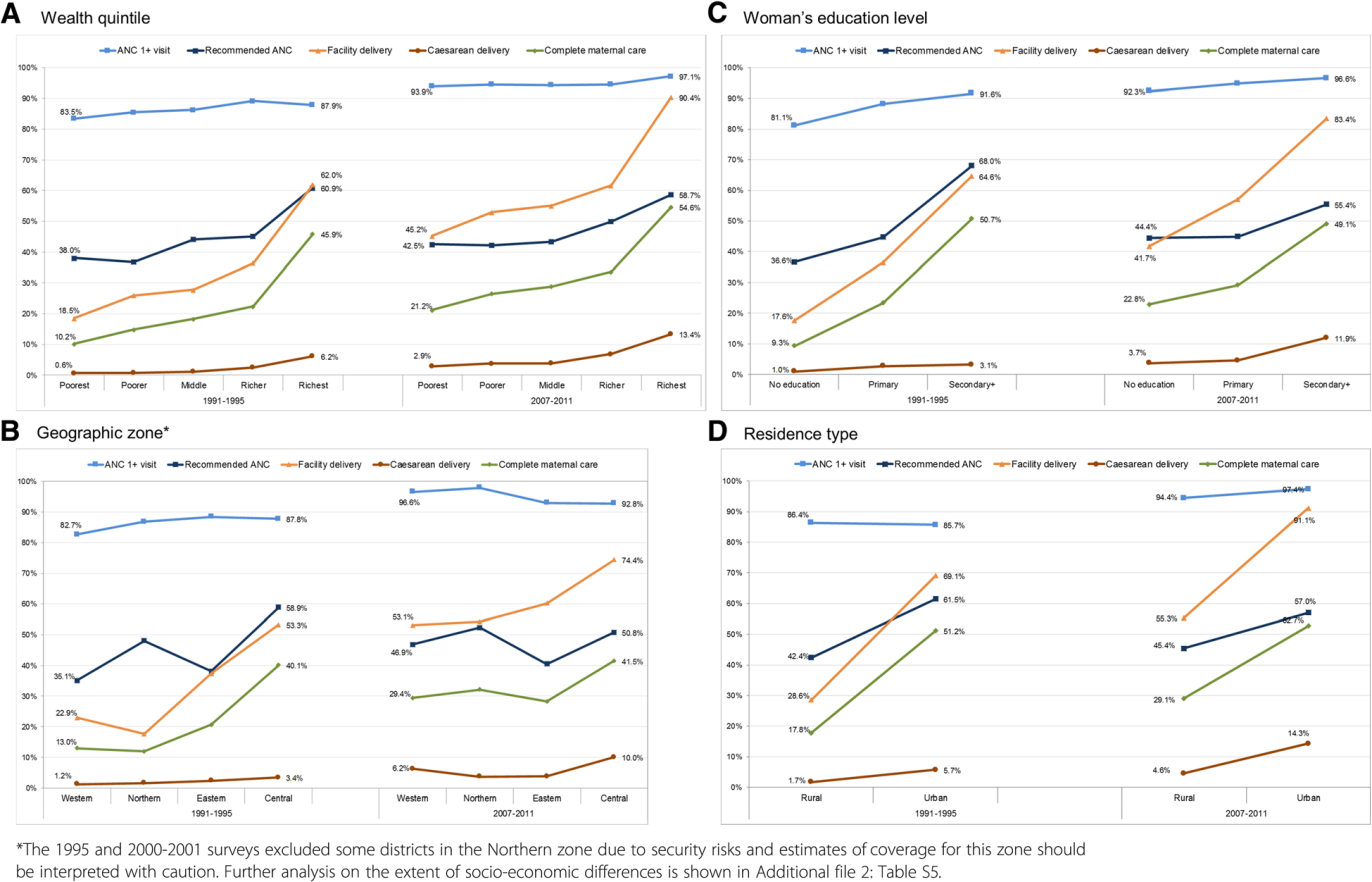
Inequalities in coverage and co-coverage indicators, earliest and most recent survey, by wealth quintile (**a**) geographic zone (**b**) woman’s education level (**c**) and residence type (**d**)

We assessed the equity of coverage (any ANC, recommended ANC, facility delivery care, caesarean section) and co-coverage (complete care) between the earliest and most recent surveys (Fig. 3). Overall, the extent of differences based on wealth, education and residence appears to have declined over time, but remained significant for all indicators in 2007–2011 (*p* < 0.010, Additional file 2: Table S5). Socio-economic differences were particularly wide for delivery care, recommended ANC, and consequently also the complete care, and for caesarean section where the differences increased over time. Having any ANC was the most equitable indicator due to high coverage across all socio-economic groups; facility delivery was least equitable. The Central zone (which includes the capital Kampala) showed the highest coverage for most indicators in all time periods. While inequities across geographic zones narrowed over time, we noted substantial differences in coverage and co-coverage between urban and rural dwellers.

### Contribution of the public and private sectors to coverage of ANC and delivery care

**Fig. 4:**
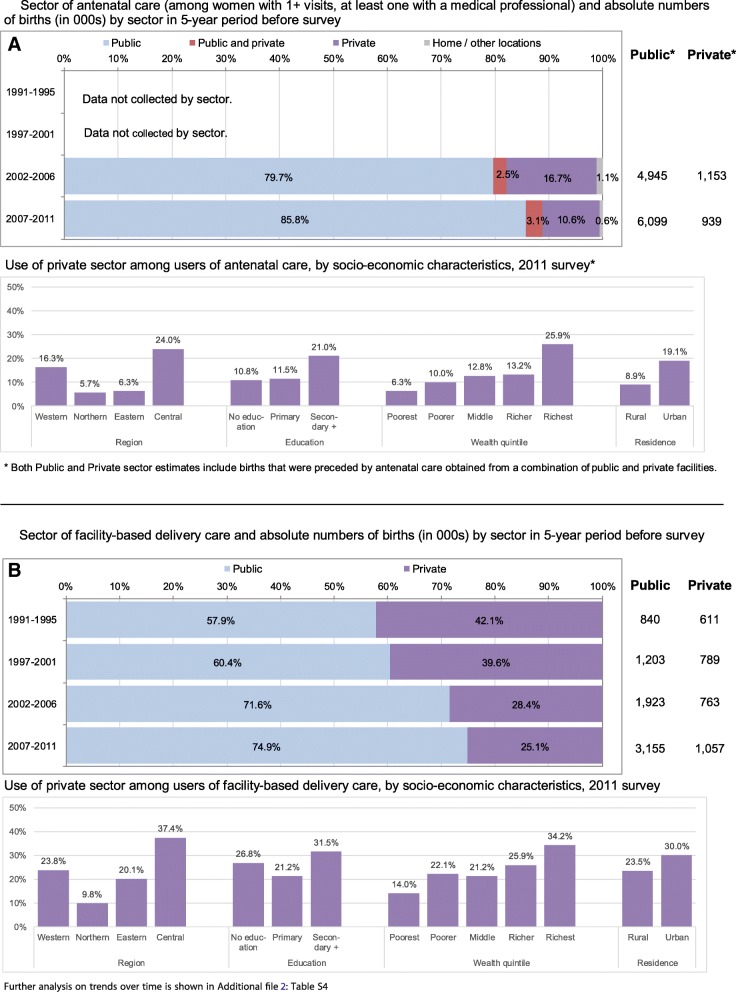
Use of health care by source among users of ANC (**a**) and delivery care (**b**) and socioeconomic differences in private sector use for the most recent birth in recall period, 2011 survey

The use of private providers among women with any ANC declined significantly from 19.2% (2006) to 13.7% in 2011 (*p* < 0.001, Fig. 4 and Additional file 2: Table S4). In the 2007–2011 period, an estimated 6.1 million women received ANC from the public sector and 0.9 million from the private sector (declining from 1.15 million in the 2002–2006 period). Figure 4 also shows that among users of facility delivery care, the proportion of women using private facilities declined significantly from 42.1% (1995) to 25.1% (2011). In 2007–2011, the public sector provided care for 3.2 million deliveries and the private sector for 1.1 million. This was an absolute increase for both sectors, from 0.9 million (public) and 0.6 million (private) in 1991–1995. Women from richest households, with secondary and higher education, residing in urban areas and in the Central zone were most likely to use private sector facilities for ANC and delivery care.

### Content of ANC and delivery care

**Fig. 5:**
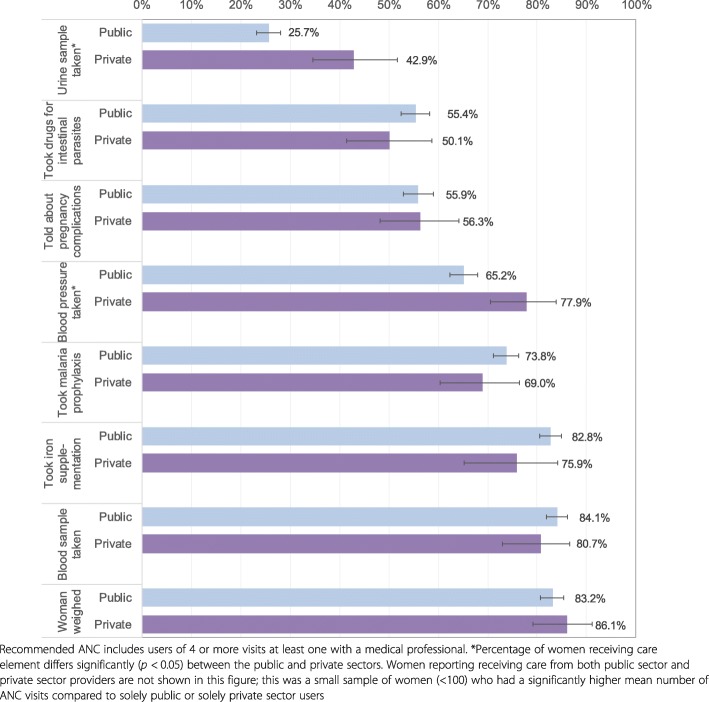
Percentage of users of recommended ANC reporting receiving content of ANC components by sector, most recent birth in recall period 2011 DHS

We examined the content of ANC and delivery care as proxies for understanding quality of care and compared the two sectors using data from the most recent survey. Figure 5 shows the percentage of women with recommended ANC who reported receiving each component, by sector of provision. The lowest overall coverage was for having a urine sample taken (27.9%), drugs for intestinal parasites (54.7%) and counselling on pregnancy complications (56.0%); the highest for iron supplementation (81.9%), having a blood sample taken (83.6%) and having been weighed (83.6%). Coverage differed significantly between the public and the private sectors for two components: having had blood pressure and urine sample taken (both were higher among users of private providers). Overall, women who received recommended ANC reported receiving on average 4.8 of the eight components; 4.9 among public and 4.2 among private sector users (not statistically different *p* > 0.05). However, 9.6% of women with recommended ANC reported receiving all eight ANC care components; this proportion was significantly higher (*p* = 0.002) for women using the private sector (17.5%) compared to those using public sector providers (8.5%).

**Fig. 6:**
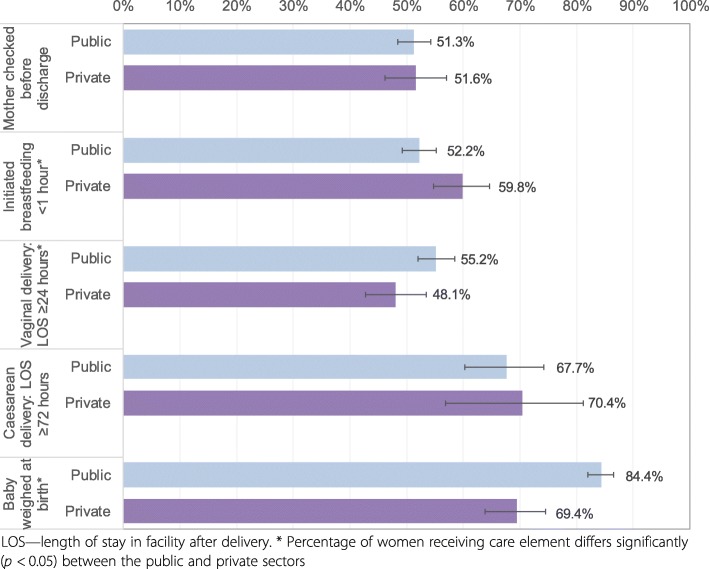
Percentage of users of facility-based delivery care reporting receiving content of delivery and postpartum care components, most recent birth in recall period 2011 DHS

Four components of delivery care were assessed among women who delivered in a facility. The coverage of each component ranged from 51.4% (checked before discharge) to 80.8% (baby weighed at birth). Figure 6 shows that significantly higher proportions of women delivering in public facilities reported that their baby was weighed at birth and that they stayed for ≥24 h following a vaginal delivery. A significantly higher proportion of women delivering in private facilities reported initiating breastfeeding within an hour of birth compared to public facilities. Overall, women reported receiving on average 2.5 of the four components (2.5 in public and 2.3 in private sector; not statistically different); with 13.0% of women delivering in health facilities receiving all four components (12.6% in public and 14.2% in private sector, difference not statistically significant).

## Discussion

This is the first study to comprehensively describe the coverage and content of ANC and delivery care, equity of coverage, and roles of the public and private sectors to these two services over time in Uganda. Over the 20-year study period, coverage with any ANC remained near universal. However, it was of insufficient frequency (among women with any ANC, fewer than half reported receiving four or more visits), perhaps partly due to late initiation. Nearly two-thirds of deliveries in 2007–2011 occurred in health facilities. However, co-coverage of ANC and delivery care was suboptimal; for example, in 2007–2011 one in three (2.6 million) babies were not born in a facility even though their mothers received some ANC. The policy on user fee removal was introduced in 2001 and the most substantial decrease in private sector delivery care utilisation occurred in the period between the 2001 and 2006 surveys. However, the significant rise in facility deliveries occurred only 5-10 years later, between the 2006 and 2011 surveys. This might be related to increased financing of primary care due to savings in the national budget arising from debt relief granted to Uganda as part of the Highly Indebted poor countries initiative in the early 2000s [46], which also enabled education and recruitment of additional health workers into government health units during the period 2005 to 2007.

Other studies have shown a rise in outpatient care-seeking following fee removal but a limited impact on inpatient services [47]. However, we showed that while the percentage of pregnant women receiving recommended ANC remained stagnant, facility delivery care rose by 37% between the 2006 and 2011 surveys. Issues that might have impeded higher utilisation of ANC and delivery care may have included physical distance from services, low capacity of facilities to absorb additional patients, concerns about indirect or unofficial costs [48–50], fear of maltreatment/neglect, lack of familial support with care-seeking, and inadequacies in staff training and referral systems [51, 52].

The caesarean section rate remained below the 5% population-level minimum recommended by WHO in two zones (Eastern and Northern) in 2011, showing that even if we assume that all caesarean sections were performed on women that needed them (and the socio-economic gradients in caesarean section rates show that this is probably not the case), there is likely to be unmet need for emergency obstetric care. As with other studies, we found that while significant inequalities in coverage by wealth, education, residence and geographic zone remained, coverage improved for all indicators among the lowest socio-economic groups of women over time [53–55]. Nevertheless, in spite of the decentralization set in motion in 2000, certain regions clearly still require additional resources to bolster their health services. Identifying and reaching the most vulnerable women with care of good quality is necessary for reaching SDGs and universal coverage.

Our study contributes to the small body of literature on the private sector in Uganda, showing that the private sector provided 14% of ANC and 25% of delivery care in 2011. To accelerate progress towards global health targets, health policymakers and program managers in Uganda have become increasingly interested in strategies to engage the private sector [30, 32, 33, 56]. Some evidence suggests that the number of private sector health facilities in Uganda is substantial and their market share may be increasing [30, 48, 49, 57]. However, we found that while the estimated number of births that occurred within the private sector increased by 73% between 1995 and 2011, the private sector market share for delivery actually declined. More dramatically, both the private sector ANC market share and number of pregnancies covered declined over the same period. This discrepancy between the literature and our findings might be due to the composition and types of services offered in the private sector, given that the majority of private sector sources in Uganda are informal and do not offer maternal health services such as ANC and delivery care, which require substantial investment in human resources and equipment/supplies [30, 35]. Uganda DHS response options only allow for a general differentiation of sector of provision into public and all others (private), but many facilities run by non-governmental and faith-based organisations are closely aligned with or subcontracted by the government and the health policy allows for a dual-sector system in which a government facility can simultaneously operate a public, free wing and a private, high-cost wing [30, 53, 58]. Our findings on the decline of private sector share have been echoed by a study of newborn care in eastern Uganda [59] and therefore it seems unlikely that this result is entirely a consequence of women’s inability to differentiate provider types.

Even when women sought maternal care, services were frequently of suboptimal quality. We found that in 2011, only one in 10 women who received recommended ANC and a similar proportion of women delivering in health facilities reported receiving all measured care components. Content of ANC and delivery care requires significant improvement in both sectors. Coverage of the individual components of care was highly variable and challenging to interpret because seemingly low resource-intensive components, such as counselling on maternal complications, had lower coverage than more demanding services, such as taking and testing blood samples. It is possible that differences relate to the role of some components in other better-funded programmes, for example the need for blood tests for programmes to reduce mother-to-child transmission of HIV [60]. It might also be related to training and communication skills of ANC providers and time constraints during consultations. A 2013 survey in four *Saving Mothers, Giving Life* districts in Uganda reported low provider obstetric knowledge and clinical confidence, and recently delivered women had concerns about availability of drugs, equipment, cleanliness and providers’ communication skills [61]. Research in Uganda and elsewhere has shown that health workers are frequently caught in binds of underperformance related to a lack of support, agency and the feeling of being valued that may, for example, contribute to lower quality ANC content [62, 63].

The population of Uganda has grown considerably over the 20-year period covered by this study, and this growth is expected to continue in the near future [3]. To maintain the same coverage levels for ANC and delivery care, the Ugandan health system has had to provide services to 30,000 additional births annually. The preliminary findings of the 2016 DHS suggest that the coverage of maternal care has increased further since the 2011 survey (any ANC: 97.3%, 4+ ANC visits: 59.9%, and facility delivery: 73.4%) [64]. Our equity analysis highlighted that future increases in ANC and delivery care toward universal coverage will necessitate reaching poor, less educated and rural women. Such strategy requires an in-depth understanding of care provision (including alternative models such as group ANC) [65] and women’s reasons for non-use of facility-based maternal care [66]. Additionally, analyses of care content and socio-economic inequities in maternal care based on the 2016 DHS will be crucial to understanding the current maternal health provision landscape and priorities in Uganda.

### Limitations

This study has a number of limitations, largely related to the nature of the DHS data used. First, all analyses relied on women’s recall of details about the care that they received for a birth that could have occurred up to 5 years prior to the date of the interview. Women’s responses were classified into pre-specified survey response options, and the direction/extent of possible bias in their recall has not been assessed in Uganda. In other countries it has been noted that women might over-report receiving content of ANC components due to social desirability [67]. Second, our analyses were affected by data availability. For instance, survey responses did not allow for disaggregation of births locations by level of facility (e.g., tertiary versus lower-level) and sources of ANC and delivery care into private for-profit and private not-for-profit (such as non-governmental and faith-based organisations) facilities. Data were not collected about the care received by women who experienced other outcomes such as miscarriages, stillbirths, and induced abortions.

In terms of ANC coverage and content, we only had information about ANC service-seeking for the most recent birth, which prevented us from deriving coverage estimates for each calendar year. We did not have details about the cadre of professional and care components for each ANC visit separately. The inability to look at ANC visits separately may have masked important patterns in service seeking and delivery. Furthermore, qualitative research from Uganda suggests that even if women predominantly use private ANC providers, they tend to make at least one visit to the public sector to receive an ANC book and other complimentary items. This means that we may have overestimated the proportion of women who used only private sector providers for ANC and that in fact, users of the combination of public and private services account for a larger proportion of provision. While we were able to examine the coverage of caesarean deliveries, we decided not to include this indicator in our analysis of content of care because the data provide no information about obstetric need for this type of delivery. Despite these limitations, this study has provided the most in-depth analysis of population-level ANC and delivery coverage and content in Uganda from 1991 to 2011.

## Conclusions

This study showed that the vast majority (> 96%) of women surveyed on the DHS in 2011 had some contact with the health system during pregnancy or delivery care. However, most women who accessed maternal services in Uganda received care with insufficient frequency, timing, continuity and content - gaps that could explain the persistently high maternal mortality levels. Our study also highlighted a decline in the share of ANC and delivery care from private sector providers, which might be connected to increased affordability of publicly-funded facilities.

Uganda’s maternal health journey over the last two decades has important implications for global policymakers. While impressive numbers of women in low- and middle-income countries have been reached by predominantly publicly-provided ANC and delivery services [68, 69], the road ahead is long, and the destination not clearly in focus. Global maternal health indicators and milestones are being re-defined, shifting the goalposts for coverage [70]. The 2016 WHO ANC guidelines recommend eight ‘contacts’ instead of four ‘visits’ [38]. Policy-makers in Uganda and in other low- and middle-income countries must therefore decide how to allocate limited financial and human resources in regard to prioritising coverage, equity or quality. For example, whether to provide women who already receive four ANC visits with four more visits (the easiest way to achieve high coverage of the new ANC guideline), or whether to address the low utilisation of ANC among poor and marginalised women with fewer than three ANC visits (and many with none). High numbers of contacts do not necessarily equate with high quality of care, and there are wide disparities across low-resource settings in receipt of essential care components among women who seek care [71]. Likewise, the lack of a single, well-defined, evidence-based, easy to measure indicator of appropriate intrapartum care is impeding progress in addressing gaps in care quality that could lead to further reductions in maternal and newborn mortality and morbidity.

## Key messages

**A: Antenatal and delivery care coverage, co-coverage and equity**

- Coverage of any ANC, facility delivery and caesarean sections significantly increased in Uganda between 1991 and 2011, which is commendable given high population growth (3% annually).
- While coverage of recommended ANC and facility delivery was strongly socio-economically patterned, gradients disadvantaging poor, less educated and rural women attenuated somewhat between 1991 and 2011.
- In 2011, 95 of 100 women had some contact with ANC providers during pregnancy, but only 33 of 100 of women received both recommended ANC and delivered in a health facility.
- In 2011, only 1 in 100 women met the 2016 WHO ANC guideline (8+ ANC contacts at least once with a medical professional and starting in the first trimester).

**B: Sector of provision**

- The share of ANC and facility delivery services obtained from the private sector (for-profit, non-governmental and faith-based organisations combined) decreased over time to 14% of ANC and 25% of delivery in 2011.
- This means that the growing numbers of births due to population rise were mainly absorbed by publicly-funded maternal care services.

**C: Content of care**

- Components of care used as proxies for care quality showed wide variation (from < 30 to > 80% of users). Many of the interventions with lowest coverage (urine sample testing, counselling on pregnancy complications, checking mother’s health before facility discharge after birth, breastfeeding initiation within 1 h of birth) do not require advanced supplies and equipment; rather they rely of sufficient staffing, frequent training and adherence to care guidelines.
- No clear differences between content of care between the public and private sectors emerged. Improvement is needed in both sectors - only 10% of ANC and 13% of delivery care users reported obtaining all measured care components in 2011.

**D: Data**

- The Demographic and Health Surveys provide a unique and valuable resource to examine time trends in maternal health utilisation and care content. Further improvements in understanding of these patterns in Uganda could be achieved by:
  - ○ Differentiating ANC and delivery care providers within the private sector further by for-profit and not-for-profit (e.g., NGO/faith-based) and by level (e.g., lower-level v tertiary).
  - ○ Providing documentation and justification on the classification of birth attendants into skilled and unskilled over time.
  - ○ Assessing the validity of women’s recall of ANC and delivery care location, cadre of medical professional, and receipt of care components.
  - ○ Collecting information on out-of-pocket expenditures for maternal care.
  - ○ Collecting data on care sought for miscarriage and stillbirths and on emergency referral pathways during delivery care.
- Women-based reports of maternal care utilisation and quality are limited and other sources of data are urgently needed, including surveys (or, ideally, censuses) of health facilities in both public and private sectors.

## Additional files

Additional file 1: **Figure S1.** Timing of Uganda’s health policies and major programmes, with focus on maternal/newborn heath. Overview of policies and programmes on a timeline (PDF document). (PDF 47 kb)

Additional file 2: **Table S1.** Sample sizes (unweighted) used in analyses, by survey. Table of sample sizes, by survey. **Table S2.** Comparison of estimates of deliveries from all births to most recent births, by survey. Table of estimates, by method. **Table S3.** Categorisation of ANC and delivery care locations into sector of provision, by survey. Table showing response options for ANC and delivery care locations by survey. **Table S4.** Change in coverage and co-coverage indicators over time. Table showing estimates and change over time. **Table S5.** Differences in the extent of socio-economic inequalities over time. Table showing extent of socio-economic inequalities. (PDF 320 kb)

## Abbreviations

ANC: Antenatal care
DHS: Demographic and Health Survey
LOS: Length-of-stay
MDG: Millennium Development Goals
MMR: Maternal mortality ratio
SBA: Skilled birth attendant
SDG: Sustainable Development Goals
WHO: World Health Organization
