# Supplementary material for: Two decades of antenatal and delivery care in Uganda: a cross-sectional study using Demographic and Health Surveys
Source: BMC Health Serv Res. 2018 Oct 4;18:758. doi: 10.1186/s12913-018-3546-3 (PMC6172797; doi:10.1186/s12913-018-3546-3)

Additional file 1: Figure S1. Timing of Uganda's health policies and major programmes, with focus on maternal/newborn health

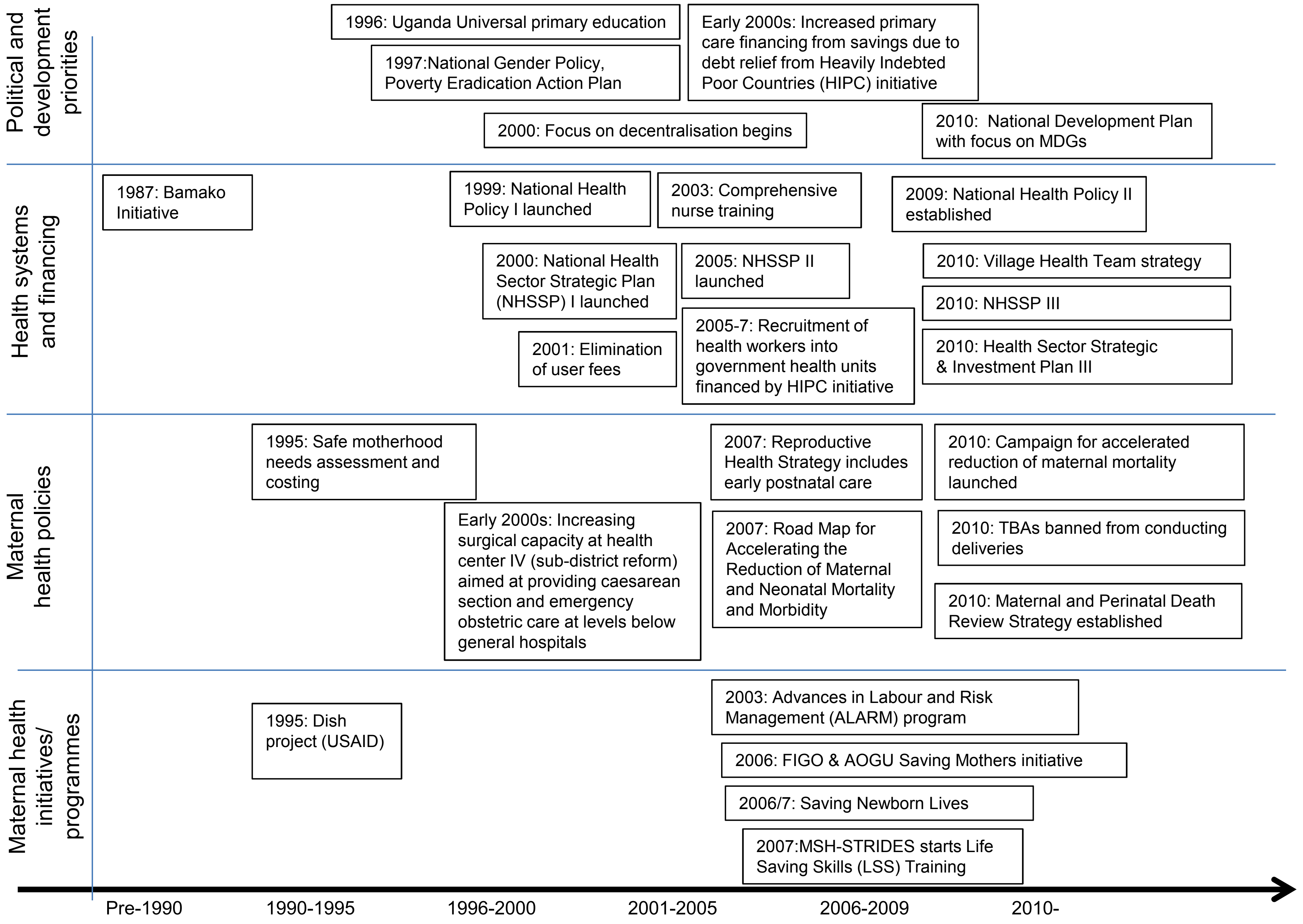

Supplement: Supplementary file 1 — Figure S1. Timing of Uganda’s health policies and major programmes, with focus on maternal/newborn heath. Overview of policies and programmes on a timeline (PDF document). (PDF 47 kb) [file 12913_2018_3546_MOESM1_ESM.pdf]
