# Supplementary material for: Two decades of antenatal and delivery care in Uganda: a cross-sectional study using Demographic and Health Surveys
Source: BMC Health Serv Res. 2018 Oct 4;18:758. doi: 10.1186/s12913-018-3546-3 (PMC6172797; doi:10.1186/s12913-018-3546-3)
Supplement: Supplementary file 2 — Table S1. Sample sizes (unweighted) used in analyses, by survey. Table of sample sizes, by survey. Table S2. Comparison of estimates of deliveries from all births to most recent births, by survey. Table of estimates, by method. Table S3. Categorisation of ANC and delivery care locations into sector of provision, by survey. Table showing response options for ANC and delivery care locations by survey. Table S4. Change in coverage and co-coverage indicators over time. Table showing estimates and change over time. Table S5. Differences in the extent of socio-economic inequalities over time. Table showing extent of socio-economic inequalities. (PDF 320 kb) [file 12913_2018_3546_MOESM2_ESM.pdf]

Additional file 2: Table S1. Sample sizes (unweighted) used in analyses, by survey

Sample description	Used in analysis presented in:	Survey year			
		1995	2001	2006	2011
All women 15-49 surveyed	-	7,070	7,246	8,531	8,674
Total number of live births in recall period	SM2	7,145	7,115	8,369	7,879
Women with 1+ live births in survey recall period (= number of most recent live births in survey recall period)	Fig 1,Fig 2, Fig 3, SM2, SM4	4,291	4,252	5,004	4,909
Women with 1+ live births in survey recall period who used 1+ ANC visits, at least one with a medical professional	Fig 4A, SM4	3,698	3,954	4,722	4,683
Women with 1+ live births in survey recall period who used 4+ ANC visits, at least one with a medical professional	Fig 5, SM4	2,015	1,895	2,377	2,447
Women with 1+ live births in survey recall period who delivered in a facility	Fig 4B, Fig 6, SM2, SM4	1,748	1,918	2,229	3,078

Additional file 2: Table S2. Comparison of estimates of deliveries from all births to most recent births, by survey

**% of births with facility delivery care**		**1995**	**2001**	**2006**	**2011**
Most recent live births in recall period	Estimate	33.6%	39.1%	44.7%	61.1%
	Lower CI	30.3%	35.5%	41.8%	58.1%
	Upper CI	37.1%	42.8%	47.7%	64.0%
All births in recall period	Estimate	29.1%	37.4%	41.3%	58.3%
	Lower CI	26.4%	34.1%	38.9%	55.4%
	Upper CI	32.1%	40.9%	43.8%	61.2%
**% births by caesarean section**		**1995**	**2001**	**2006**	**2011**
Most recent live births in recall period	Estimate	2.2%	3.0%	3.6%	6.1%
	Lower CI	1.6%	2.5%	3.0%	5.3%
	Upper CI	2.9%	3.6%	4.3%	7.1%
All births in recall period	Estimate	1.8%	2.5%	3.1%	5.3%
	Lower CI	1.4%	2.1%	2.6%	4.5%
	Upper CI	2.3%	3.0%	3.8%	6.1%

CI – 95% confidence interval

Additional file 2: Table S3. Categorisation of ANC and delivery care locations into sector of provision, by survey

<table>
<tr><th>Survey</th><th>Antenatal care locations</th><th>Delivery care locations</th></tr>
<tr><td>1995</td><td>Not captured</td><td>Public sector: government hospital, government health center, government health unit, other public sector

Private sector: Private hospital/clinic, other private medical</td></tr>
<tr><td>2000</td><td>Not captured</td><td>Public sector: government hospital, government health center, government health/aid post, other public sector

Private sector: Private hospital/clinic, other private medical</td></tr>
<tr><td>2006</td><td colspan="2">Public sector: government hospital, government health center, government health post, other public

Private sector: Private hospital/clinic, other private medical</td></tr>
<tr><td>2011</td><td colspan="2">Public sector: government hospital, government health center, other public sector

Private sector: Private hospital/clinic, other private</td></tr>
</table>

Additional file 2: Table S4. Change in coverage and co-coverage indicators over time

	Estimate (95%CI), by survey				Change over time from earliest (1995, ANC sector: 2006) to most recent survey (2011)	
**% of most recent live births in survey recall period with**	**1995**	**2001**	**2006**	**2011**	**Absolute percentage point change**	**p-value (Chi2)**
1+ ANC visits, at least one with a medical professional	86.4% (84.8-87.8)	92.4% (91.3-93.4)	94.1% (93.1-95.0)	94.9% (93.8-95.7)	8.5%	<0.001
Received in the public sector*	n/a	n/a	79.7% (77.1-82.1)	85.8% (83.7-87.6)	6.1%	<0.001
Received in the private sector	n/a	n/a	16.7% (14.5-19.2)	10.6% (9.0-12.4)	-6.1%	<0.001
Recommended ANC (4+ ANC visits, at least one with a medical professional)	44.7% (42.3-47.3)	41.6% (39.1-44.1)	46.6% (44.5-48.7)	47.3% (45.2-49.4)	2.6%	0.134
Facility delivery	33.6% (30.3-37.1)	39.1% (35.5-42.8)	44.7% (41.8-47.7)	61.1% (58.1-64.0)	27.5%	<0.001
Received in the public sector	57.9% (53.5-62.2)	60.4% (56.2-64.5)	71.6% (68.2-74.8)	74.9% (71.9-77.7)	17.0%	<0.001
Received in the private sector	42.1% (37.8-46.5)	39.6% (35.5-43.8)	28.4% (25.2-31.8)	25.1% (22.3-28.1)	-17.0%	<0.001
Caesarean section	2.2% (1.6-2.9)	3.0% (2.5-3.6)	3.6% (3.0-4.3)	6.1% (5.3-7.1)	3.9%	<0.001
Complete maternal care	21.9% (19.4-24.6)	22.7% (20.3-25.4)	26.3% (24.3-28.4)	32.9% (30.7-35.2)	11.0%	<0.001

* Public and private categories do not sum to 100% due to other two categories (public/private and home/other locations) as shown on Figure 4. The sum of these two categories remained the same between 2006 and 2011 (3.6%), therefore change over time is solely due to changes in the use of public versus private sector, as shown here.

Additional file 2: Table S5. Differences in the extent of socio-economic inequalities over time

	Earliest survey (1995)		Most recent survey (2011)	
**Socio-economic characteristics***	**Absolute percentage point difference**	**p-value (Chi2)**	**Absolute percentage point difference**	**p-value (Chi2)**
**Wealth quintile (poorest versus richest)**				
1+ ANC visits, at least one with a medical professional	4.4%	0.031	3.2%	0.007
Recommended ANC	22.9%	<0.001	16.2%	<0.001
Facility delivery	43.5%	<0.001	45.2%	<0.001
Caesarean section	5.5%	<0.001	10.5%	<0.001
Complete maternal care	35.7%	<0.001	33.4%	<0.001
**Education level (no education versus secondary and higher)**				
1+ ANC visits, at least one with a medical professional	10.5%	<0.001	4.3%	0.004
Recommended ANC	31.4%	<0.001	11.0%	<0.001
Facility delivery	47.0%	<0.001	41.7%	<0.001
Caesarean section	2.2%	0.007	8.2%	<0.001
Complete maternal care	41.4%	<0.001	26.3%	<0.001
**Residence (rural versus urban)**				
1+ ANC visits, at least one with a medical professional	-0.7%	0.668	3.0%	0.010
Recommended ANC	19.1%	<0.001	11.6%	<0.001
Facility delivery	40.5%	<0.001	35.8%	<0.001
Caesarean section	4.0%	<0.001	9.7%	<0.001
Complete maternal care	33.4%	<0.001	23.6%	<0.001

* Differences by geographic zone are not shown because categories with lowest and highest coverage are not consistently the same across time and indicators.
